# Retraction Note: A non-enzymatic, isothermal strand displacement and amplification assay for rapid detection of SARS-CoV-2 RNA

**DOI:** 10.1038/s41467-025-59976-9

**Published:** 2025-05-17

**Authors:** Mohsen Mohammadniaei, Ming Zhang, Jon Ashley, Ulf Bech Christensen, Lennart Jan Friis-Hansen, Rasmus Gregersen, Jan Gorm Lisby, Thomas Lars Benfield, Finn Erland Nielsen, Jens Henning Rasmussen, Ellen Bøtker Pedersen, Anne Christine Rye Olinger, Lærke Tørring Kolding, Maryam Naseri, Tao Zheng, Wentao Wang, Jan Gorodkin, Yi Sun

**Affiliations:** 1 Department of Health Technology, Technical University of Denmark, Lyngby, Denmark; 2 PentaBase A/S, Petersmindevej 1 A, Odense C, Denmark; 3 Department of Clinical Biochemistry, Copenhagen University Hospital - Bispebjerg and Frederiksberg, Copenhagen, Denmark; 4 Department of Emergency Medicine, Copenhagen University Hospital - Bispebjerg and Frederiksberg, Copenhagen, Denmark; 5 Department of Clinical Microbiology 445, Copenhagen University Hospital - Amager and Hvidovre, Copenhagen, Denmark; 6 Department of Infectious Diseases, Copenhagen University Hospital - Amager and Hvidovre, Hvidovre, Denmark; 7 Center for Translational Research, Copenhagen University Hospital – Bispebjerg and Frederiksberg, Copenhagen, Denmark; 8 Department of Occupational and Environmental Medicine, Copenhagen University Hospital - Bispebjerg and Frederiksberg, Copenhagen, Denmark; 9 Center for non-coding RNA in Technology and Health, Department of Veterinary and Animal Sciences, Faculty of Health and Medical Sciences, University of Copenhagen, Frederiksberg C, Denmark

Retraction to: *Nature Communications* 10.1038/s41467-021-25387-9, published online 24 August 2021

The authors have retracted this article. After publication, the authors were notified that other research groups had been unable to reproduce the findings reported in this publication. Specifically, they had found that the assay only functions as reported in this article at high concentrations of template RNA. Upon repeating their experiments the authors themselves found that the reproducibility of their results is highly dependent on multiple factors, including the manufacturing origin of the probes used and the PCR machine employed. The authors are currently investigating this dependence and aim to report their findings in due course. However, the authors no longer have confidence in the reliability of the results reported in this article.

All authors agree with this retraction.

